# The adaptation of colorectal cancer cells when forming metastases in the liver: expression of associated genes and pathways in a mouse model

**DOI:** 10.1186/s12885-017-3342-1

**Published:** 2017-05-19

**Authors:** Derya Bocuk, Alexander Wolff, Petra Krause, Gabriela Salinas, Annalen Bleckmann, Christina Hackl, Tim Beissbarth, Sarah Koenig

**Affiliations:** 1 Department of General, Visceral and Paediatric Surgery, University Medical Centre, Georg – August – University Goettingen, Göttingen, Germany; 2 Statistical Bioinformatics, Department of Medical Statistics, University Medical Centre, Georg – August – University Goettingen, Göttingen, Germany; 3 Microarray and Deep-Sequencing Core Facility, Institute for Developmental Biochemistry, University Medical Centre, Georg – August – University Goettingen, Göttingen, Germany; 4 Department of Haematology and Medical Oncology, University Medical Centre, Georg – August – University Goettingen, Göttingen, Germany; 5 Department of Surgery, University Hospital Regensburg, Regensburg, Germany; 6 Medical Teaching and Medical Education Research, University Hospital Wuerzburg, Julius-Maximilians-University Wuerzburg, Josef-Schneider-Str. 2/D6, 97080 Wuerzburg, Germany

**Keywords:** Colorectal cancer (CRC), RNA-sequencing, Gene expression, Liver metastasis

## Abstract

**Background:**

Colorectal cancer (CRC) is the second leading cause of cancer-related death in men and women. Systemic disease with metastatic spread to distant sites such as the liver reduces the survival rate considerably. The aim of this study was to investigate the changes in gene expression that occur on invasion and expansion of CRC cells when forming metastases in the liver.

**Methods:**

The livers of syngeneic C57BL/6NCrl mice were inoculated with 1 million CRC cells (CMT-93) via the portal vein, leading to the stable formation of metastases within 4 weeks. RNA sequencing performed on the Illumina platform was employed to evaluate the expression profiles of more than 14,000 genes, utilizing the RNA of the cell line cells and liver metastases as well as from corresponding tumour-free liver.

**Results:**

A total of 3329 differentially expressed genes (DEGs) were identified when cultured CMT-93 cells propagated as metastases in the liver. Hierarchical clustering on heat maps demonstrated the clear changes in gene expression of CMT-93 cells on propagation in the liver. Gene ontology analysis determined inflammation, angiogenesis, and signal transduction as the top three relevant biological processes involved. Using a selection list, matrix metallopeptidases 2, 7, and 9, wnt inhibitory factor, and chemokine receptor 4 were the top five significantly dysregulated genes.

**Conclusion:**

Bioinformatics assists in elucidating the factors and processes involved in CRC liver metastasis. Our results support the notion of an invasion-metastasis cascade involving CRC cells forming metastases on successful invasion and expansion within the liver. Furthermore, we identified a gene expression signature correlating strongly with invasiveness and migration. Our findings may guide future research on novel therapeutic targets in the treatment of CRC liver metastasis.

**Electronic supplementary material:**

The online version of this article (doi:10.1186/s12885-017-3342-1) contains supplementary material, which is available to authorized users.

## Background

Colorectal cancer (CRC) is the third most common type of cancer in the Western world and the second most common cause of cancer-related death in both genders. The overall relative 5-year survival of CRC patients is approximately 50% [1]. Almost half of all patients suffering from CRC are confronted with liver metastasis either at the time of diagnosis (15 to 20%), or later during the course of the disease (25%) [2]. Given the rather poor 5-year survival rate of patients who develop liver metastasis (approx. 30%), it is vital that we develop and evaluate new therapeutic strategies. In particular, the knowledge of molecular changes to CRC cells that end up in the liver may enable us to search for new target options far more selectively.

Metastasis is frequently a final and fatal step in the progression of solid malignancies. The nature and time of onset of the changes that provide tumour cells with metastatic functions are still largely unknown. Furthermore, there has been an ongoing debate to this end for more than a 100 years. In 1889, Stephen Paget noticed that the pattern of metastases produced by different neoplasms was not random. In his ‘seed and soil’ hypothesis, Paget claimed that certain tumour cells (‘seeds’) have an affinity for the microenvironment of specific organs (‘soil’), and only when the ‘seed’ and the ‘soil’ are compatible can metastasis occur [3].

With respect to the “seed”, it is widely accepted these days that cancer is attributed to the accumulation of genetic alterations in cells. Thus, to understand the molecular mechanisms of cancer metastasis, it is indispensable to identify not only the genes whose alterations accumulate during cancer progression but also those genes whose expression is responsible for the acquisition of metastatic potential in cancer cells [4]. Indeed, comparative analyses of the gene expression profiles of metastatic and non-metastatic cells have revealed that various genes are differentially expressed in association with the metastatic potential of cancer cells [4]. Conversely, the existence of genes expressed by rare cellular variants that specifically mediate metastasis has been disputed [5]. Transcriptomic profiling of primary human carcinomas has identified gene expression patterns that, when present in the primary tumour, predict a poor prognosis for patients [6, 7]. The existence of such signatures can be interpreted in the sense that genetic lesions acquired early on in tumorigenesis may prove sufficient for the metastatic process, and that consequently no metastasis-specific genes exist.

There is growing evidence that the development of or progression to metastases is also dependent on the “soil”. Tumour cell circulation, extravasation into a distant organ, angiogenesis, and uninhibited growth also provide essential hints as to the metastatic process [8]. The molecular requirements for some of the steps involved may be highly tissue specific. For example, the proclivity that tumours have for specific organs, such as breast carcinomas for bone and lung, was noted more than a century ago [9]. Moreover, the potential of tumour cells to metastasize depends on their interaction with homeostatic factors in the target organ that promote tumour-cell growth: survival, angiogenesis, invasion, and progression. It seems that the intrinsic cellular heterogeneity within tumour populations evolves through an extrinsic selection process, which is based on more or less infrequent cellular variants with augmented metastatic abilities and which finally mediates the outgrowth in distant sites [9]. Of note, the mechanism that enables the liver microenvironment to influence the behaviour of CRC cells is still only poorly understood.

The most common site for CRC metastasis is the liver [10]. Many patients still suffer from recurrence of the primary and/or distant metastasis, even after undergoing liver resection combined with adjuvant approaches such as chemo- and radiotherapy. Nonetheless, only a minority of patients actually survive for years [11]. Therefore, the a priori or early inhibition of metastasis could prove to be a key step towards the curative treatment of patients. We have to assume that each organ places different demands on circulating cancer cells for the homing and subsequent outgrowth of metastases.

To clarify this issue, we established a novel syngeneic and orthotopic mouse model of CRC liver metastasis. This model comprises the injection of cells from a known CRC cell line to mimic the spread of the primary tumour and thus to investigate the invasion and expansion of CRC cells in the liver on the gene expression level. The goal here was to identify genes that contribute to this process of adapting to the new “soil” and thereby the metastatic progression of the disease. The fundamental aim of the study was to identify new candidate markers or molecular mechanisms in the diagnosis of liver metastasis resulting from CRC, as well as therapeutic targets effectively inhibiting CRC metastasis in the liver.

## Methods

### Reagents and antibodies

Unless specified otherwise, all chemicals and reagents were supplied by Life Technologies (Darmstadt, Germany). Foetal Bovine Serum Superior (FBS) was purchased from Biochrom (Berlin, Germany) and trypsin 10-fold was supplied by PAA (Pasching, Austria). Antibodies for immunolabelling purposes were purchased and used as illustrated in Table 1.

**Table 1:** Antibodies used in immunolabelling analysis

Antigen	Species	Dilution	Catalogue	Manufacturer
β-catenin	Rabbit	1:50	14–6765	eBioscience, Frankfurt a.M., Germany
CD44	Rat	1:1000	550,538	BD Pharmingen, Heidelberg, Germany
Ki-67	Rabbit	1:200	275R-14	Cell Marque, California, United States
E-cadherin	Rabbit	1:50	sc-7870	Santa Cruz Biotechnology, Heidelberg, Germany
Vimentin	Rabbit	1:1000	ab92547	Abcam, Cambridge, UK
Anti-rat biotinylated	Donkey	1:200	RPN1004	GE Healthcare, Freiburg, Germany
Avidin HRP		1:400	18–4100-94	eBioscience, Frankfurt a.M., Germany
HRP Labelled anti-rabbit	Goat	Ready to use	K4002	Dako, Hamburg, Germany

### Cell lines and culture

The cell line CMT-93 (isolated from a mouse colorectal adenocarcinoma) was kindly donated by Christina Hackl and her workgroup in Regensburg, Germany. On testing, the cells were found to be negative for mycoplasma by RT-PCR.

CRC cells were expanded and stored in frozen aliquots (− 70 °C). After thawing, the cells were routinely cultured in 75 cm^2^ culture flasks in DMEM high glucose, supplemented with 10% FBS, 1% L-glutamine, 1% sodium pyruvate and 1% penicillin/streptomycin at 37 °C and 5% CO_2_ in a humidified incubator. Tumour cells were passaged once (following 3 days in culture), cultured for a further 4 days, and then trypsinized for subsequent implantation studies. Tumour cells from the same passage were used for all the implantation experiments. Additionally, aliquots of the cell line were snap frozen and processed for transcriptome sequencing analysis (RNA-seq).

### Animals and procedures

Ten-week-old female C57BL/6NCrl mice (mass 18–22 g) were purchased from Charles River (Sulzfeld, Germany). Animals were kept on a 12-h day/night rhythm and fed with a phytoestrogen-reduced mouse diet (ssniff, Soest, Germany).

Prior to (surgical intervention) surgery, animals received a subcutaneous application of carprofen (Rimadyl®, Pfizer, Berlin, Germany) (5 mg/kg body mass). Animals were anaesthetized under constant sevoflurane inhalation (Sevorane®, Abbott, Wiesbaden, Germany). After median laparotomy, the hilum of the liver was exposed to access the portal vein. One million tumour cells in a volume of 100 μl PBS buffer were injected slowly into the portal vein using a 30 G needle.

In the study group, seven animals were implanted with tumour cells. The control group encompassed five animals which underwent the same procedures (sham-OP), but were only injected with buffer solution. All animals were sacrificed after 4 weeks. Explanted livers were sliced for macroscopic assessment, photographic documentation of the section planes, and further processing. Tissue samples from the tumour core of the liver metastases derived from CMT-93 as well as matched unharmed liver tissue (macroscopically tumour-free liver) were excised, snap frozen for whole transcriptome sequencing analysis (RNA-seq), or frozen in 2-methylbutane at −70 °C for immunolabelling.

### Immunolabelling

Cryostat sections (5 μm) were fixed in ice-cold acetone for 10 min and were stored at −80 °C. After rehydration in Tris/HCl buffer (pH 7.6), sections were incubated with the primary antibodies (see Table 1) overnight at 4 °C. Endogenous peroxidase was inactivated by incubation with 0.3% H_2_O_2_ in 70% methanol and 30% Tris/HCl buffer for 20 min at RT. The HRP-labelled goat anti-rabbit IgG secondary antibody (DakoCytomation K4002, Carpinteria, USA, ready-to-use reagent) was used to identify β-catenin, Ki-67, E-cadherin, and vimentin. To immunolabel CD44, sections were exposed to an avidin/biotin blocking step (Life Technologies, Darmstadt, Germany) followed by incubation with the primary antibody (overnight at 4 °C). This antigen was identified by the secondary antibodies donkey anti-rat biotinylated (1:200, 1 h at RT) and avidin-horseradish peroxidase (HRP) (1:400, 1 h at RT). 3-amino-9-ethyl-carbazole (AEC) solution (BD Pharmingen, Heidelberg, Germany) and haematoxylin counterstaining were used for visualization by light microscopy. Negative controls were carried out for each antibody by omitting the primary antibody from the protocol. Samples were covered with 50 μl of the aqueous mounting agent Aquatex (Merck, Darmstadt, Germany) and evaluated under a light microscope (LEICA DM IRE2, Bensheim, Germany).

### RNA isolation

For RNA sequencing purposes (RNA-seq), three aliquots of the cell line and specimens (tumour core and liver) from seven animals were collected. The RNA purification system PeqGold TriFast (Peqlab, Erlangen, Germany) was used to isolate RNA from metastatic liver tissue. Briefly, specimens were defrosted in peqGold TriFast (1 ml/100 mg tissue) and then homogenized using TissueLyser LT (Qiagen, Hilden, Germany) at 50 Hz. Total RNA was isolated according to the manufacturer’s instructions and stored at −80 °C. In addition, the High Pure RNA Isolation Kit (Roche, Grenzach-Wyhlen, Germany) was used to isolate RNA from CMT-93 cells according to the manufacturer’s recommendations. The quantity and integrity of the isolated RNA was assessed in a NanoDrop ND − 1000 spectrophotometer, version 3.5.2 (Peqlab, Erlangen, Germany), using the 260 nm/280 nm absorbance ratio and was further analysed with an Agilent 2100 BioAnalyzer (Agilent Technologies, Santa Clara, California, USA) as a quality check. RNA-seq was performed at the Transcriptome and Genome Analysis Laboratory in Goettingen, Germany, using an Illumina HiSeq2000 sequencer (Illumina, Inc., San Diego, California, USA).

### Deep sequencing analysis

As starting material for the library preparation, 0.5 μg of total RNA was used. The libraries were generated according to the TruSeq mRNA Sample Preparation Kits v2 Kit from Illumina (Cat. N°RS − 122-2002). The fluorometric based QuantiFluor™ dsDNA System from Promega (Mannheim, Germany) was used for accurate quantitation of cDNA libraries. The size of final cDNA libraries was determined by using the Fragment Analyzer from Advanced Bioanalytical. cDNA libraries were amplified and sequenced by using the cBot and HiSeq2000 from Illumina (SR; 1 × 50 bp; ca. 30 Mio reads per sample). Sequence images were transformed to bcl files using Illumina software BaseCaller, which were demultiplexed to fastq files with CASAVA v1.8.2 and quality checks were done via fastqc.

### Statistics

#### Preparation of data/statistical model

An in-house RNA-seq analysis pipeline employing the STAR-aligner (version 2.4.0 h) [12] for the mapping and counting of reads with the expectation-maximization algorithm implemented in the software package RSEM (version 1.2.19) [13] was used for counting reads. Ensembl *Mus musculus* GRCh38 Version 78 was considered as the reference for mapping and further annotations.

Following RNA-seq, all seven tumour probes derived from CMT-93 underwent quality control measures. Employing the corresponding RNA-seq data, they were checked for the expression of CK 20 as surrogate parameter for colorectal tissue or liver-specific gene expression to identify liver-specific genes, such as phosphoenolpyruvate-carboxykinase 1 (PCK1), cytochrome p450 (CYP), and carbamoyl phosphate synthase1 (CPS1). Three tumour probes (D213K, D214K, D215K) representing false biopsies were excluded from further analysis owing to a strong infiltration of liver (approx. 4 to 20 times the elevated expression levels of liver enzymes) and low content of colorectal tissue.

Principal component analysis (PCA) was performed in R, the programming language and environment (version 3.2), to visualize the underlying structure of the dataset by calculating the eigenvectors and plotting those two components with the highest variance in the data.

Focussing on the comparison between the cell line and metastases, we filtered out differential genes specific to liver tissue, which we considered as ‘liver tissue effect’. Thus, differentially expressed genes (DEGs) were identified as either up- or down-regulated when comparing the CMT-93 cell line with the unharmed liver tissue. Subsequently, these differences relating to the normal liver background were excluded from the gene expression results between the cell line and liver metastases. This filtering step was done in order to identify genes representing differences in cell line versus tumour, instead of general differences in cell lines versus normal liver.

#### Significant differential gene analysis

Basing on the read counts attained from RSEM, the R package EdgeR [14] was used to calculate the mean intensities as well as the *p*-value and the log fold change (logFC) for each DEG, comparing the CMT-93 cells with the liver metastases formed. Thus, gene differences between the two groups were identified by fitting a negative binomial generalized linear model implemented in EdgeR. Expression results were reported as mean transcripts per million (TPM) values for each group.

A list was created comprising 119 genes associated with metastasis, based on the genes described in the Tumor Metastasis RT2 Profiler PCR Array by Qiagen Hilden, Germany (Additional file 1). This list was applied as a filter following completion of the analysis of the DEGs to profile the expression of these genes in our dataset.

#### Gene ontology (GO) analysis

A gene set was defined, comprising all the DEGs identified in the comparison of CMT-93 cells and the liver metastases that formed, corrected for the liver background and with a false discovery rate of less than 5% (FDR < 0.05). This gene set was employed in the gene ontology and pathway analysis. This method, implemented in the R package topGO [15], allows us to identify GO terms that are over-represented (or under-represented) using the annotations for that gene set taken from the Gene Ontology Database (http://www.geneontology.org/). The significant level of GO terms for the DEGs was analysed with the weighted Fisher’s exact test in the package. We computed *p*-values for all the DEGs in the GO category “biological processes”; the threshold of significance was defined as *p*-value <0.05.

## Results

### Syngeneic mouse model of CRC metastasis in the liver

**Fig. 1:**
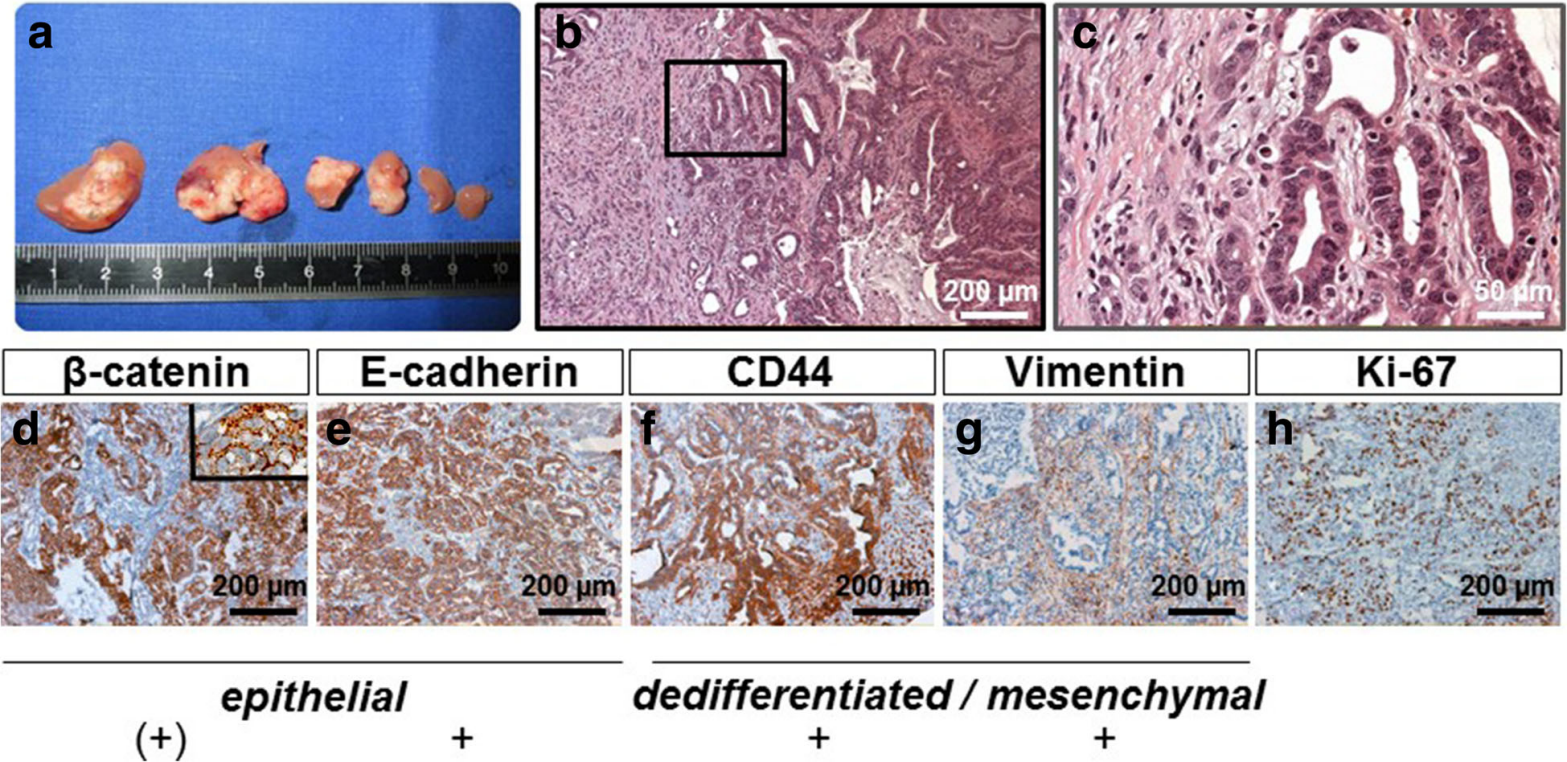
Macroscopic and microscopic aspects of liver metastases derived from CMT-93. Macroscopic overview of metastatic growth 4 weeks following implantation of CMT-93 cells via the portal vein in syngeneic C57BL/6NCrl mice (treatment group). Explanted livers were sliced for macroscopic assessment (**a**) and subjected to H&E staining (**b, c**). Liver metastases represented a moderately differentiated colorectal adenocarcinoma revealing complex glandular structures (50–75% gland formation) in a desmoplastic stroma. Subpanel **d** depicts the results of immunolabelling to detect ß-catenin (the inlay illustrates the mostly cytoplasmic location of expression) and **e** confirms E-cadherin, both markers indicative of the glandular structures. The dedifferentiated/mesenchymal differentiation was identified by staining with anti-vimentin (**g**) and anti-CD44 (**f**). Ki-67 was used as a proliferation marker as illustrated in **h** Scales are as indicated

Following the injection of CMT-93 cells via the portal vein and their subsequent expansion, 70–80% of animals developed liver metastases in any number of liver lobules during the course of the study, as seen on macroscopic assessment (Fig. 1a). Within the life span of the animals, CMT-93 cells had colonized about 30–50% of the mouse liver with the tumour spots increasing to approximately 5 to 10 mm in diameter. However, the spread of the tumour burden resulting from CMT-93 proved to be inhomogeneous when comparing the right and left liver lobules of injected mice. On the microscopic level, immunohistochemical staining 4 weeks after tumour cell injection into the portal vein was used to assess phenotypic expression of colorectal carcinoma markers. Liver metastases displayed features of a moderately differentiated colorectal adenocarcinoma revealing complex glandular structures (40–75% gland formation) in a desmoplastic stroma (Fig. 1 b, c). An important feature of invasion is the presence of this desmoplasia or desmoplastic reaction, a type of fibrous proliferation surrounding tumour cells and secondary to the invasive tumour growth. The glandular structures expressed epithelial markers such as membrane-bound β-catenin (Fig. 1d) and E-cadherin (Fig. 1e). The latter was expressed mostly in the cytoplasm, which indicates that this wnt marker was inactive. The hyaluronic acid receptor CD44 (a putative marker of ‘stemness’ in CRC) was also present and staining was detected in nearly all of the tumour cells we investigated (Fig. 1f). The unsystematic arrangements of gland formations also expressed the mesenchymal marker vimentin (Fig. 1g). The proliferation marker Ki-67 was expressed abundantly in more than 75% of all liver tumours in a random pattern (Fig. 1h).

### Adaptation of CMT-93 cells when forming metastases in the liver

**Fig. 2:**
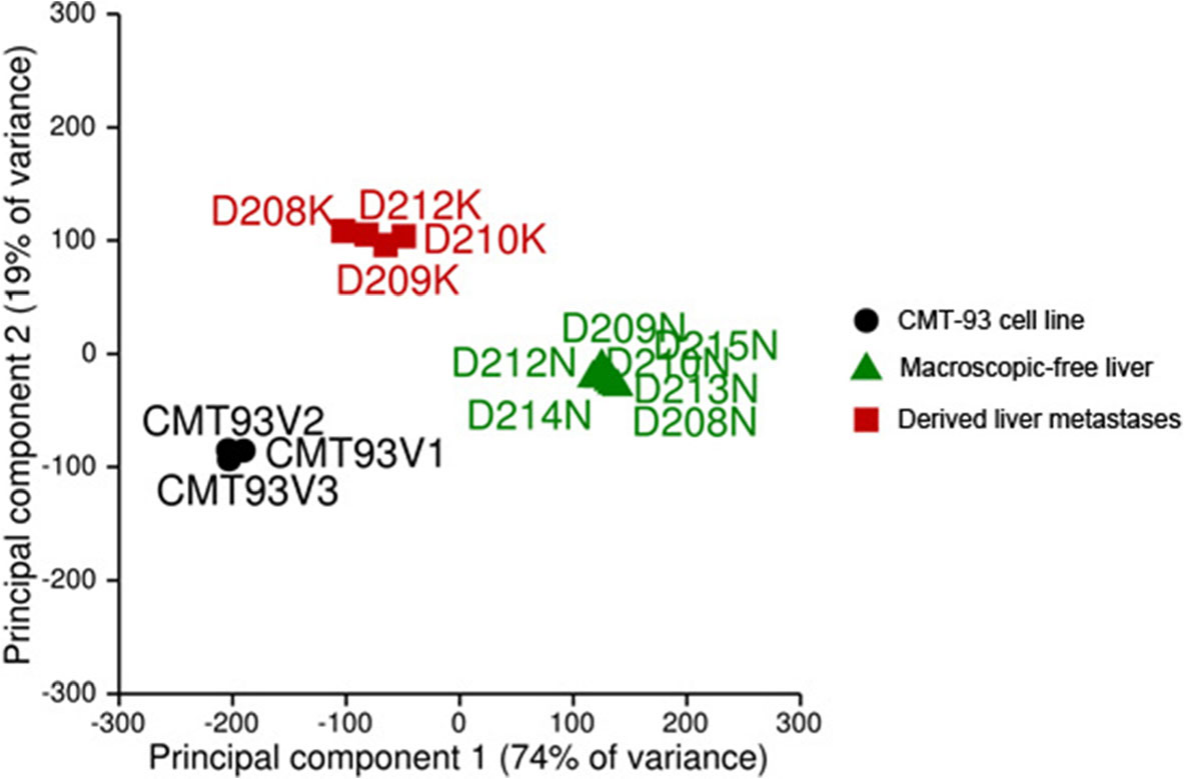
PCA was used to visualize the underlying structure of the dataset. CMT-93 cells as well as tumour probes (*tumour liver tissue*) formed clusters clearly separate from the corresponding macroscopically tumour-free liver. Each group was tightly clustered

To assess the factors involved in the formation of liver metastases, we performed RNA-seq analysis on both the liver metastases as well as unaffected liver tissue. Figure 2 summarizes the structure of the gene expression data. The first principal component (PC) is plotted on the x-axis and captures 74% of the variance. The second PC is plotted on the y-axis and captures 19% of the variance. The PCA plot clearly portrays the separation of the CMT-93 cell line samples from those of the corresponding macroscopically tumour-free liver, as well as from the derived liver metastases. As assumed, the cluster associated with CMT-93 was found to be located in close proximity to the cluster relating to the derived metastases. We also plotted liver specimens originating from sham-operated animals (injection of buffer alone). When overlaid on the PCA plot in Fig. 2, these samples lay in exactly the same position as the cluster of the macroscopically tumour-free liver samples (data not illustrated).

Table 2 lists the DEGs during the propagation of CMT-93 cells in the liver. A total of 5297 genes were down-regulated and 6597 were up-regulated when CMT-93 cells propagated in the liver. The elimination of DEGs relating to the liver background (see materials and methods) reduced the total number of DEGs to 1174 down-regulated genes (35%) and 2155 up-regulated genes (65%).

**Table 2:** Differences in gene expression among the sample groups (DEGs)

	CMT-93 vs. liver metastases	Macroscopic-free liver vs. liver metastases	CMT-93 vs. macroscopic-free liver	CMT-93 vs. liver metastases corrected for the liver background
−1 = down-regulated	5297	5163	5764	1174
0 = unregulated	3667	4157	2684	0
1 = up-regulated	6597	5749	6187	2155

**Fig. 3:**
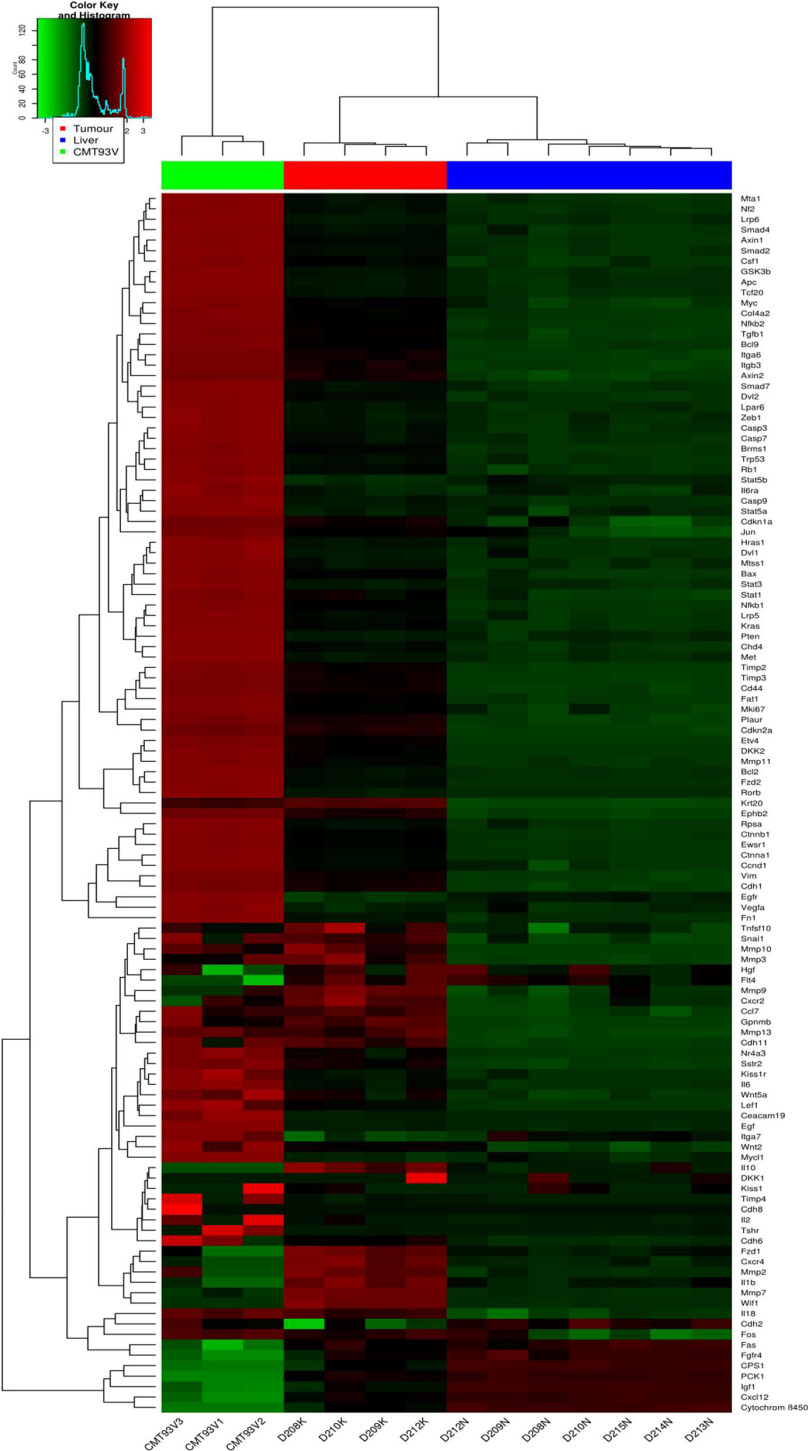
Top 120 DEGs between CMT-93 cells, liver metastases derived from CMT-93 and macroscopically tumour-free liver. Expression data are depicted as a data matrix in which each row represents a gene and each column represents a sample. The colour coding *bar above the heat map marks* the samples from the CMT-93 cell line (samples CMT93V1–3) as *green*, those from the liver metastases derived from CMT-93 (samples D208-210 K, D212K) in *red* and those of macroscopically tumour-free liver (samples D208-210 N, D212-215 N) in *blue*. Expression levels are depicted according to the *colour scale* presented in the *top left* corner. *Red* indicates expression levels *above* and *green* *below* the median, respectively. The magnitude of deviation from the median is represented by the colour saturation. The hierarchical clustering is visualized by the dendrogram at the *top*, which illustrates the degree of relatedness in gene expression

The results of hierarchical cluster analysis to assess the relatedness of the 120 DEGs displaying the greatest differences in expression are presented in Fig. 3. The heat map reveals systematic and fairly clearly distinguished variations in the expression of genes between the original CMT-93 cells, the derived liver metastases, and tumour-free liver. Of note, the changes in expression of the CMT-93 cells outgrowing as liver metastases is clearly apparent. However, the hierarchical clusters relating to the metastases and the tumour-free liver are somewhat closer to each other owing to the fact that the implanted CMT-93 cells forming metastases infiltrate the liver tissue. The gene expression profile is therefore bound to reflect the colonization of the hepatic tissue.

**Fig. 4:**
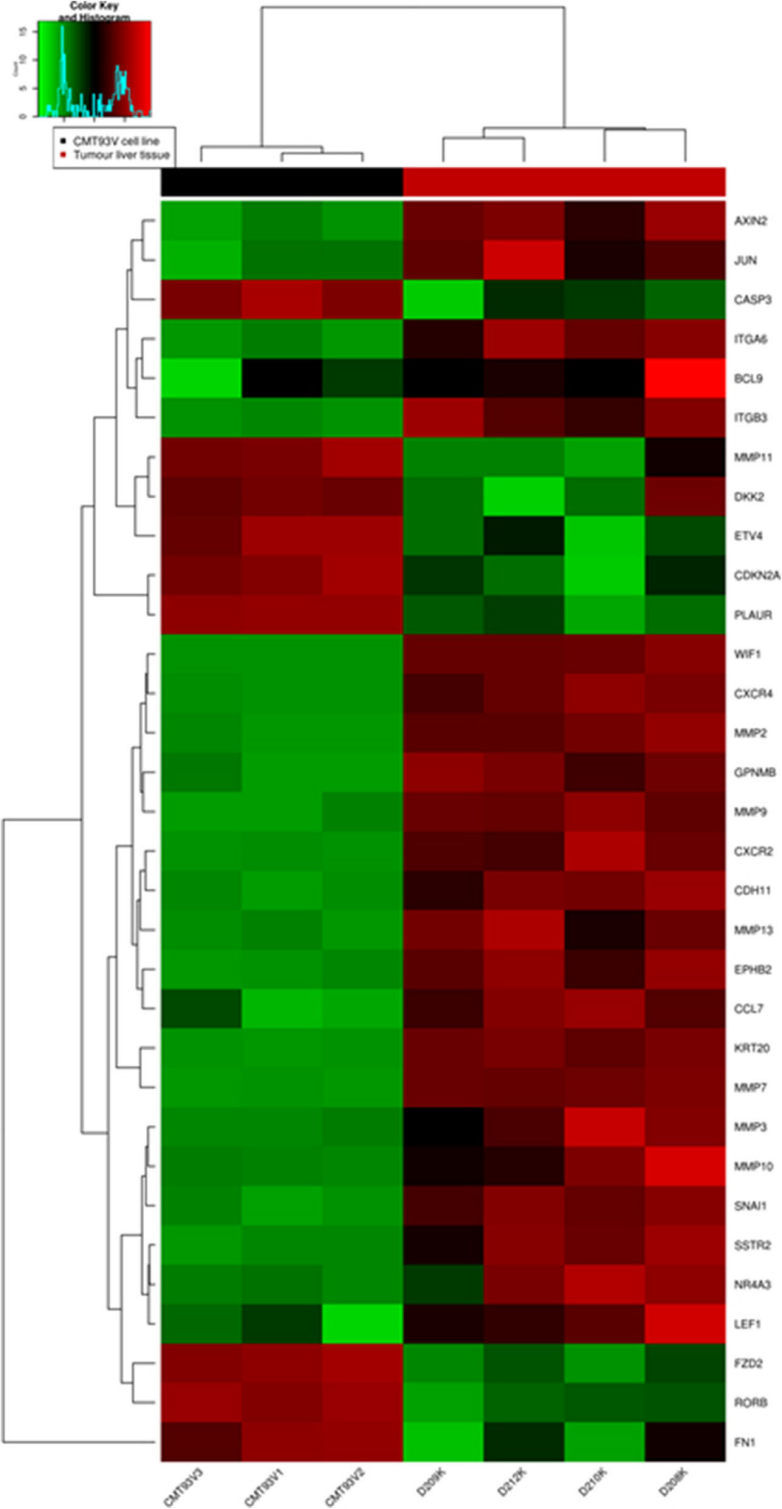
Heat map illustrating the expression signature of 32 genes related to liver metastasis development. Unsupervised analysis was performed on the data set using our filtered gene list (119 genes associated with metastasis). Depicted are 32 representative genes which were expressed differentially during the propagation of CMT-93 cells in the liver

We then applied a filter set of 119 selected genes associated with metastasis to our RNA-seq data set following the elimination of DEGs determining the liver background. Thus, 32 relevant genes were identified with a threshold of FDR < 5%, of which 23 were up-regulated and 9 down-regulated. Additional file 2 contains the count data of the samples. Figure 4 illustrates the heat map of the filtered DEGs within the CMT-93 cell line and the liver metastasis samples. Hierarchical clustering confirmed the clear changes between DEGs of the CMT-93 cells and the derived metastases. Table 3 presents an overview of these genes which were also ranked by *p*-value. The top five dysregulated genes were found to be matrix metallopeptidase (MMP) 7, keratin 20 as an epithelial marker of colorectal carcinoma, wnt inhibitory factor 1, MMP 9, and chemokine receptor 4.

**Table 3:** Overview of the most relevant genes associated with metastasis during liver colonisation (FDR < 5%)

Gene	Ensembl ID	Gene Symbol	Functional Gene Group	*p*-value	Mean intensity CMT-93	Mean intensity liver metastases	logFC*	logFC^	FDR
Matrix metallopepti-dase 7	ENSMUSG00000018623	MMP7	Extracellular Matrix Proteins	1,7 × 10^−180^	0,02	72,56	8,97	11,69	4,9 × 10^−178^
Keratin 20	ENSMUSG00000035775	KRT20	CRC-Related Genes	2,9 × 10^−174^	0,81	51,99	5,8	6,12	7,7 × 10^−172^
Wnt inhibitory factor 1	ENSMUSG00000020218	WIF1	Wnt Signalling (canonical)	3,7 × 10^−126^	0,00	20,68	7,37	12,85	3,3 × 10^−124^
Matrix metallopepti-dase 9	ENSMUSG00000017737	MMP9	Extracellular Matrix Proteins	3,8 × 10^−72^	0,10	14,16	5,95	7,92	9 × 10^−71^
Chemokine (C-X-C motif) receptor 4	ENSMUSG00000045382	CXCR4	Cell Growth and Proliferation Genes	1,5 × 10^−71^	0,02	19,29	7,06	10,50	3,4 × 10^−70^
Matrix metallopepti-dase 2	ENSMUSG00000031740	MMP2	Extracellular Matrix Proteins	1,8 × 10^−70^	0,09	27,83	7,04	8,33	4,2 × 10^−69^
Eph receptor B2	ENSMUSG00000028664	EPHB2	Cell Growth and Proliferation Genes	4,8 × 10^−59^	1,06	10,86	3,2	3,65	8 × 10^−58^
Chemokine (C-X-C motif) receptor 2	ENSMUSG00000026180	CXCR2	Cell Growth and Proliferation Genes	1,1 10^−40^	0,04	7,53	5,51	7,75	9,7 × 10^−40^
Cadherin 11	ENSMUSG00000031673	CDH11	Cell Adhesion Genes	8,8 × 10^−40^	0,12	6,32	4,67	5,75	7,9 × 10^−39^
Axin2	ENSMUSG00000000142	AXIN2	Wnt Signalling (canonical)	2,2 × 10^−39^	20,31	72,10	1,82	1,87	2 × 10^−38^
Integrin beta 3	ENSMUSG00000020689	ITGB3	Cell Adhesion Genes	1,6 × 10^−33^	5,54	25,48	2,17	2,35	1,2 × 10^−32^
Matrix metallopeptidase 13	ENSMUSG00000050578	MMP13	Extracellular Matrix Proteins	8,1 × 10^−29^	0,22	7,45	4,43	5,20	4,8 × 10^−28^
Glycoprotein (transmembrane) nmb	ENSMUSG00000029816	GPNMB	Cell Adhesion Genes	3,9 × 10^−27^	0,21	14,50	5,45	6,25	2,1 × 10^−26^
Snail family zinc finger 1	ENSMUSG00000042821	SNAI1	EMT Transition	2,9 × 10^−26^	0,12	3,05	3,62	4,66	1,5 × 10^−25^
Plasminogen activator, urokinase receptor	ENSMUSG00000046223	PLAUR	Cell Growth and Proliferation Genes	8,9 × 10^−20^	191,38	71,50	−1,42	−1,28	3,6 × 10^−19^
Matrix metallopeptidase 3	ENSMUSG00000043613	MMP3	Extracellular Matrix Proteins	3,7 × 10^−17^	0,05	2,70	3,95	5,74	1,3 × 10^−16^
Integrin alpha 6	ENSMUSG00000027111	ITGA6	Cell Adhesion Genes	5 × 10^−17^	12,03	31,23	1,36	1,32	1,8 × 10^−16^
Matrix metallopeptidase 10	ENSMUSG00000047562	MMP10	Extracellular Matrix Proteins	7,6 × 10^−17^	0,06	2,68	3,83	5,37	2,7 × 10^−16^
Somatostatin receptor 2	ENSMUSG00000047904	SSTR2	Cell Growth and Proliferation Genes	5,7 × 10^−15^	0,22	1,57	2,19	3,17	1,8 × 10^−14^
Jun proto-oncogene	ENSMUSG00000052684	JUN	Transcription Factors and Regulators	7,8 × 10^−13^	22,13	44,89	1,01	1,16	2,3 × 10^−12^
Frizzled homolog 2 (Drosophila)	ENSMUSG00000050288	FZD2	Wnt Signalling (canonical)	1,1 × 10^−12^	6,88	2,16	-1,7	-1,54	3 × 10^−12^
Chemokine (C-C motif) ligand 7	ENSMUSG00000035373	CCL7	Cell Growth and Proliferation Genes	2,4 × 10^−12^	0,98	9,98	3,18	3,47	6,9 × 10^−12^
Nuclear receptor subfamily 4, group A, member 3	ENSMUSG00000028341	NR4A3	Transcription Factors and Regulators	3,5 × 10^−12^	0,16	1,22	2,1	3,31	1 × 10^−11^
RAR-related orphan receptor beta	ENSMUSG00000036192	RORB	Cell Growth and Proliferation Genes	2,6 × 10^−11^	2,57	0,70	-1,94	-1,68	7 × 10^−11^
Cyclin-dependent kinase inhibitor 2A	ENSMUSG00000044303	CDKN2A	Transcription Factors and Regulators	1,9 × 10^−8^	179,73	91,40	−0,98	−0,84	4,4 × 10^−8^
Matrix metallopeptidase 11	ENSMUSG00000000901	MMP11	Extracellular Matrix Proteins	6,6 × 10^−7^	12,30	5,36	−1,21	−1,19	1,4 × 10^−6^
Ets variant 4	ENSMUSG00000017724	ETV4	Transcription Factors and Regulators	9,5 × 10^−7^	21,60	10,13	−1,1	−0,93	1,9 × 10^−6^
Lymphoid enhancer binding factor 1	ENSMUSG00000027985	LEF1	EMT Transition	5,3 × 10^−5^	0,37	1,19	1,26	1,69	9,3 × 10^−5^
Caspase 3	ENSMUSG00000031628	CASP3	Apoptosis Genes	0,00027	48,58	29,55	−0,72	−0,57	0,00045
Fibronectin 1	ENSMUSG00000026193	FN1	EMT Transition	0,00085	1051,50	805,60	−0,38	−0,74	0,00134
B cell CLL/lymphoma 9	ENSMUSG00000038256	BCL9	Wnt Signalling (canonical)	0,00222	15,50	19,20	0,3	0,42	0,00336
Dickkopf homolog 2 (*Xenopus laevis*)	ENSMUSG00000028031	DKK2	Wnt Signalling (canonical)	0,02671	9,31	6,03	−0,65	−0,50	0,03566

LogFC* describes changes between/comparing the mean intensities calculated as the log fold changes of base 2 referring to the TPM-values, logFC^ represents the log fold change of base 2 from the R package EdgeR

With respect to functional gene groups, the 23 up-regulated genes were attributed to extracellular matrix proteins (6 genes), cell growth and proliferation (5 genes), cell adhesion (4 genes), wnt signalling (3 genes), transcription factors/regulators and epithelial-mesenchymal transition (EMT) (2 genes each), and CRC-related genes (1 gene). The 9 down-regulated genes were assigned to cell growth and proliferation, wnt signalling and transcription factors/regulator (2 genes each), as well as apoptosis genes, extracellular matrix proteins, and EMT (1 gene each).

### Biological processes involved

**Fig. 5:**
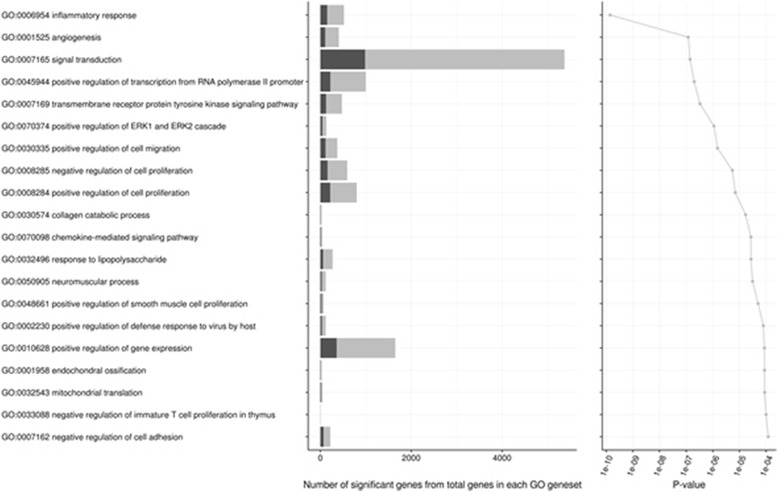
Bar plot of GO pathways. Top 20 Gene Ontology categories from biological processes comparing the CMT-93 *cell line* with the liver metastases derived thereof. GO categories are depicted and sorted by *p*-value. The *dark grey bar* indicates the number of significant genes from all genes in that GO category (*dark and light grey*). Together with the *light grey* region of the bar, the plot summarises the total number of genes on the pathway

To address the pathways and processes involved, significant DEGs with FDR < 5% were selected and tested against the background set of all genes with GO annotation (Fig. 5). The most relevant GO terms for biological processes enriched were “inflammatory response”, “angiogenesis”, “signal transduction”, “positive regulation of transcription from RNA polymerase II promoter”, “transmembrane receptor protein tyrosine kinase signalling pathway”, and “positive regulation of ERK1 and ERK2 cascade”.

## Discussion

CRC is a common disease whose considerable metastatic potential highlights the urgency and necessity to develop novel therapeutic approaches to prevent or treat tumour progression and metastasis. In this study, we set out to analyse the changes in gene expression and pathways that play a role in the colonisation of mouse liver by the cell line CMT-93, mimicking the processes that lead to the ultimate formation of liver metastases secondary to CRC. To this end, we first had to establish the in vivo metastatic mouse model. CMT-93 cells demonstrate a strikingly efficient tumorigenic capacity following their implantation via the portal vein, the common route CRC cells take when colonising the liver. Furthermore, the CMT-93 cell line originated from the mouse strain we employ, C57BL/6NCrl; the two are thus syngeneic and this enables us to circumvent rejection responses as complications. Moreover, the mice are immunocompetent, which allows us to investigate the normal inflammatory response to tumour growth. Of note, the outgrowing metastases in this model are reproducible and display a number of prototypic features (structure and markers) common to human CRC liver metastases. The model itself acts as a paradigmatic proof of principle for genetic alteration when forming liver metastases. A xenograft model involving human CRC cell lines would not have been able to fulfil our criterion of immunocompetence to mimic the seed and soil theory in humans. While searching for suitable models we tried three combinations in total, based on literature research and commercial availabilities. Although CT-26 (ATCC® CRL-2639™) and the mouse strain Balb/c resulted in tumour formation in the liver, these were found to be mesenchymally de-differentiated in nature. The cell line APC1638-NT (kindly donated by Prof. R. Smits, Rotterdam, Netherlands) with mouse strains C57BL/6 N or C57BL/6 J resulted in no detectable tumour growth whatsoever (data not shown). The CMT-93/C57BL/6NCrl model proved to be the only one demonstrating reproducible liver metastatic growth with the histological features of colorectal cancer.

Disseminating tumour cells need to adapt to surrounding tissues in a continuous fashion [16]. For example, CRC cells of the primary tumour have to avoid succumbing to any immune response, detach from the primary, migrate into the portal system, arrive in the liver, traverse the endothelial barrier of the portal vessels (extravasation), overcome hypoxia on integration into the liver parenchyma, adapt to the new environment, initiate angiogenesis, and finally expand as metastases (metastatic colonisation) [17–19]. Taken together, these numerous influences during the process of metastatic spread into clinically detectable macroscopic disease lead to marked changes in gene expression. This new gene signature is the result of a multi-step process in which carcinoma cells progress along the “invasion-metastasis cascade” [18]. The results of our study visibly support the notion that CRC cells certainly undergo a number of clear changes in the liver environment.

It goes without saying that every single finding from the dataset following RNA-seq analysis cannot be commented on in this highly specific context during propagation in the liver. However, there are some definite hints as to which genes and pathways have to be addressed when aiming to treat CRC liver metastasis.

Our RNA-seq expression analysis and subsequent filtering with the selection list of the most-relevant metastasis-related genes reveals that the liver environment stimulates CMT-93 cells into expressing a number of genes enhancing metastasis. These genes are associated with functions such as tissue remodelling, cell proliferation, adhesion, wnt activity, transcription/regulation, and inhibition of apoptosis, which all contribute to metastatic activity and tumour cell invasion. Specifically, MMPs, chemokine receptors, and integrins are predominantly up-regulated in liver metastases derived from CMT-93.

Interactions between carcinoma cells and stromal cells play a vital role during the invasion of the new anatomic metastatic site. Tumour cells must first traverse the basement membrane and then create space for further expansion. Components of the extracellular matrix (ECM) contain a repository of growth factor molecules that can be liberated by proteases secreted by carcinoma tissue. Moreover, the basement membrane also plays crucial roles in signal transduction events within carcinoma cells via pathways initiated by integrin-mediated cell-matrix adhesions, leading to alterations in cell polarity, proliferation, invasiveness, and survival [20]. Additionally, the entry of CRC cells into the hepatic microvasculature can also initiate the pro-inflammatory cascade that results in Kupffer cells being triggered to secrete chemokines [21]. Those are known to up- and also down-regulate various vascular adhesion receptors, thereby enabling adhesion of CRC cells in the microvasculature of the fibroblasts and myofibroblasts, endothelial cells, adipocytes, and various bone-marrow-derived cells – including macrophages and other immune cells [22].

GO analysis is widely recognized as the premier tool in the organization and functional annotation of molecular aspects of cellular systems [23]. We determined the significant GO categories based on a threshold of significance of *p* < 0.05. Our results reveal that the GO terms inflammatory response, angiogenesis, and signal transduction were the most relevant biological processes involved in the propagation of CRC in the liver. Most recently, Becht et al. reported that CRC molecular subgroups and micro-environmental signatures were highly correlated [24]. More precisely, he stated that the mesenchymal subtype of CRC was characterized by a high density of fibroblasts that most likely produces the chemokines and cytokines which favour tumour-associated inflammation and support angiogenesis, resulting in a poor prognosis. Looking into features of assessable inflammatory state in patients, Hamilton et al. were able to link elevated serum levels of C - reactive protein (CRP) with increased circulating pro-inflammatory cytokines. Those patients with colorectal liver metastases were attributed with shorter disease-free and overall survival following surgical resection [25]. Most recently, the inflammatory milieu of CRC liver metastases was used to investigate a new treatment option based on TIE2-expressing monocytes/macrophages (TEMs), a myeloid cell subset. Adopting the concept of gene transfer, TEMs located in peritumoral sites and exerted an anti-tumour effect through the release of interferon-alpha (IFNα) [26]. Utilizing this strategy in mouse models of CRC liver metastasis, TEMs accumulate in the proximity of hepatic metastatic areas and the TEM-mediated delivery of IFNα inhibits tumour growth. In our study, we could not detect IFNα as being deregulated to a significant level in the liver metastases. However, there were a number of DEGs associated with interferon (e.g. interferon-activated gene 205 (IFI205), interferon-induced transmembrane protein 1 (IFITM1) and interferon gamma inducible protein 30 (IFI30)), which were not members of the top 100 list (data not shown).

Different angiogenic factors have been related to metastasis formation because they promote primary tumour growth and increase the likelihood that tumour cells come into contact with blood and thus disseminate [27]. In particular, the liver is known to be a permissive soil with respect to angiogenesis. The liver parenchyma adjacent to the synchronous liver metastases provides an angiogenically favourable environment for metastatic tumour growth [28]. On the individual level, there was significant correlation between primary CRCs and matched liver metastases with respect to vascular endothelial growth factor (VEGF) mRNA expression. VEGF mRNA levels in patients with two or more liver metastatic tumours were significantly higher than those in patients with only solitary liver metastases [29]. To date, oxaliplatin- and irinotecan-based chemotherapy regimens combined with monoclonal antibody treatment in the form of bevacizumab (anti-VEGF) have proved to be efficient as first-line therapy of metastatic colorectal cancer [30, 31].

Invasion processes are crucial to the formation of liver metastases in CRC and regularly involve a variety of MMPs leading to the degradation and remodelling of the extracellular matrix (ECM) [18, 27]. CRC liver metastases express MMP7 more intensely than normal liver [32]. Our results support the notion that MMP7 is one of the significant players enhancing invasiveness in CRC [33–35]. It is worth noting here that targeted therapy in this matter is difficult. There is evidence in some pre-clinical models that MMP inhibitors (MMPIs) are effective at multiple stages of CRC tumour progression, inhibiting both establishment and growth of primary CRC tumours, as well as reducing metastasis in the lungs and liver [36]. However, clinical trials with MMPIs have been largely unsuccessful as therapeutic agents in CRC so far. A recent study in pre-clinical mouse models of metastatic CRC suggests that ulinastatin (an intrinsic trypsin inhibitor) and natural polyphenol curcumin are capable of inhibiting CRC liver metastases via modulation of MMP9 and E-cadherin expression [37].

The model developed certainly proved to be suitable, as the invasion and expansion of CMT-93 following their injection via the portal vein leading to liver metastasis was reproducible. Our results clearly support the notion of an invasion-metastasis cascade with notable changes to the expression profile of CMT-93 cells on entering and expanding in the liver. Although we were perhaps able to shed some light on the bigger picture, the relative importance of distinct events, interactions, and the molecular drive that all serve to facilitate organ-specific colonisation will require further investigation.

## Conclusions

Our work demonstrates that the gene expression in tumour cells is clearly altered during and following the process of metastasis. Here, bioinformatics greatly assists in the analysis of large amounts of data derived from RNA-seq. Through rigorous experimental planning and sophisticated statistical analysis, we are a step closer to elucidating the factors and processes involved during the liver metastasis of CRC. One or more of these dysregulated genes may prove to be a worthy target and enable us effectively to switch off a CRC cell’s capacity to act as seed in the formation of metastases. Such a development, at best during the early stages of disease progression, for example prior to the outgrowth of tumour cells within the target soil, could well have a markedly positive effect on the prognosis as well as overall survival of CRC patients.

## Additional files

Additional file 1: Supplement 1. List of 119 genes associated with metastasis. The dataset was filtered using this list to identify DEGs between the CMT-93 cell line and liver metastases derived from CMT-93. Gene names and Ensembl IDs are shown in Additional file 1: Supplement 1. (XLSX 11 kb)

Additional file 2: Supplement 2. Count data of relevant genes for the propagation of CMT-93 cells in the liver. Thirty-two DEGs were identified with a threshold of FDR < 5%, of which 23 were up-regulated and 9 down-regulated. Gene names, count data and Ensembl IDs are shown in Additional file 2: Supplement 2. (XLSX 191 kb)

## Abbreviations

AEC: 3-amino-9-ethyl-carbazole
BMBF: German Ministry of Education and Research
CPS1: Carbamoyl phosphate synthase1
CRC: Colorectal Cancer
CRP: C-reactive protein
CYP: Cytochrome p450
DEG: Differentially expressed genes
ECM: Extracellular matrix
EdgeR: Empirical Analysis of Digital Gene Expression Data in R
EMT: Epithelial-mesenchymal transition
FBS: Foetal bovine serum
FDR: False discovery rate
GO: Gene ontology
HRP: Horseradish peroxidase
IFI205: Interferon-activated gene 205
IFI30: Interferon gamma inducible protein 30
IFITM1: Interferon-induced transmembrane protein 1
IFNα: Interferon-alpha
LogFC: Log fold change
MMP: Matrix metallopeptidase
MMPIs: MMP inhibitors
PBS: Phosphate-buffered saline
PC: Principal component
PCA: Principal component analysis
PCK1: Phosphoenolpyruvate-carboxykinase 1
R: Programming language
RSEM: RNA-seq by Expectation-Maximization
RT: Room temperature
TEMs: TIE2-expressing monocytes/macrophages
TPM: Transcripts per million
VEGF: Vascular endothelial growth factor

## References

[1] Machii R, Saika K (2014). Five-year relative survival rate of colon cancer in the USA, Europe and Japan. Jpn J Clin Oncol.

[2] Leporrier J, Maurel J, Chiche L, Bara S, Segol P, Launoy G (2006). A population-based study of the incidence, management and prognosis of hepatic metastases from colorectal cancer. Br J Surg.

[3] Paget S (1989). The distribution of secondary growths in cancer of the breast. Cancer Metastasis Rev.

[4] Yokota J (2000). Tumor progression and metastasis. Carcinogenesis.

[5] Bernards R, Weinberg RA (2002). A progression puzzle. Nature.

[6] Ramaswamy S, Ross KN, Lander ES, Golub TR (2003). A molecular signature of metastasis in primary solid tumors. Nat Genet.

[7] Lim B, Mun J, Kim JH, Kim CW, Roh SA, Cho DH, Kim YS, Kim SY, Kim JC (2015). Genome-wide mutation profiles of colorectal tumors and associated liver metastases at the exome and transcriptome levels. Oncotarget.

[8] Chambers AF, Groom AC, MacDonald IC (2002). Dissemination and growth of cancer cells in metastatic sites. Nat Rev Cancer.

[9] Talmadge JE, Fidler IJ (2010). AACR centennial series: the biology of cancer metastasis: historical perspective. Cancer Res.

[10] Clark ME, Smith RR (2014). Liver-directed therapies in metastatic colorectal cancer. J Gastrointest Oncol.

[11] Mi K, Kalady MF, Quintini C, Khorana AA (2015). Integrating systemic and surgical approaches to treating metastatic colorectal cancer. Surg Oncol Clin N Am.

[12] Dobin A, Davis CA, Schlesinger F, Drenkow J, Zaleski C, Jha S, Batut P, Chaisson M, Gingeras TR (2013). STAR: ultrafast universal RNA-seq aligner. Bioinformatics.

[13] Li B, Dewey CN (2011). RSEM: accurate transcript quantification from RNA-Seq data with or without a reference genome. BMC Bioinformatics.

[14] Robinson MD, McCarthy DJ, Smyth GK (2010). edgeR: a Bioconductor package for differential expression analysis of digital gene expression data. Bioinformatics.

[15] Alexa A, Rahnenfuhrer J (2010). topGO: topGO: enrichment analysis for Gene ontology. R Packag Version.

[16] Al-Taee KK, Ansari S, Hielscher T, Berger MR, Adwan H (2014). Metastasis-related processes show various degrees of activation in different stages of pancreatic cancer rat liver metastasis. Oncol Res Treat.

[17] Fidler IJ (2003). The pathogenesis of cancer metastasis: the 'seed and soil' hypothesis revisited. Nat Rev Cancer.

[18] Valastyan S, Weinberg RA (2011). Tumor metastasis: molecular insights and evolving paradigms. Cell.

[19] Gupta GP, Massague J (2006). Cancer Metastasis: building a framework. Cell.

[20] Bissell MJ, Hines WC (2011). Why don't we get more cancer? A proposed role of the microenvironment in restraining cancer progression. Nat Med.

[21] Wen SW, Ager EI, Christophi C (2013). Bimodal role of Kupffer cells during colorectal cancer liver metastasis. Cancer Biol Ther.

[22] Joyce JA, Pollard JW (2009). Microenvironmental regulation of metastasis. Nat Rev Cancer.

[23] Lovering RC, Camon EB, Blake JA, Diehl AD (2008). Access to immunology through the Gene ontology. Immunology.

[24] Becht E, de Reynies A, Giraldo NA, Pilati C, Buttard B, Lacroix L, Selves J, Sautes-Fridman C, Laurent-Puig P, Fridman WH. Immune and stromal classification of colorectal cancer is associated with molecular subtypes and relevant for precision immunotherapy. Clin Cancer Res. 2016;22(16):4057–66. 10.1158/1078-0432.CCR-15-2879 26994146

[25] Hamilton TD, Leugner D, Kopciuk K, Dixon E, Sutherland FR, Bathe OF (2014). Identification of prognostic inflammatory factors in colorectal liver metastases. BMC Cancer.

[26] Catarinella M, Monestiroli A, Escobar G, Fiocchi A, Tran NL, Aiolfi R, Marra P, Esposito A, Cipriani F, Aldrighetti L (2016). IFNalpha gene/cell therapy curbs colorectal cancer colonization of the liver by acting on the hepatic microenvironment. EMBO Mol Med.

[27] Nadal C, Maurel J, Gascon P (2007). Is there a genetic signature for liver metastasis in colorectal cancer?. World J Gastroenterol.

[28] van der Wal GE, Gouw AS, Kamps JA, Moorlag HE, Bulthuis ML, Molema G, de Jong KP (2012). Angiogenesis in synchronous and metachronous colorectal liver metastases: the liver as a permissive soil. Ann Surg.

[29] Kuramochi H, Hayashi K, Uchida K, Miyakura S, Shimizu D, Vallbohmer D, Park S, Danenberg KD, Takasaki K, Danenberg PV (2006). Vascular endothelial growth factor messenger RNA expression level is preserved in liver metastases compared with corresponding primary colorectal cancer. Clin Cancer Res.

[30] Kocakova I, Melichar B, Kocak I, Bortlicek Z, Buchler T, Dusek L, Petruzelka L, Kohoutek M, Prausova J, Finek J (2015). Bevacizumab with FOLFIRI or XELIRI in the first-line therapy of metastatic colorectal carcinoma: results from Czech observational registry. Anticancer Res.

[31] Yamazaki K, Nagase M, Tamagawa H, Ueda S, Tamura T, Murata K, Eguchi Nakajima T, Baba E, Tsuda M, Moriwaki T, et al. Randomized phase III study of bevacizumab plus FOLFIRI and bevacizumab plus mFOLFOX6 as first-line treatment for patients with metastatic colorectal cancer (WJOG4407G). Ann Oncol. 2016;27(8):1539–46. 10.1093/annonc/mdw206 27177863

[32] Zeng ZS, Shu WP, Cohen AM, Guillem JG (2002). Matrix metalloproteinase-7 expression in colorectal cancer liver metastases: evidence for involvement of MMP-7 activation in human cancer metastases. Clin Cancer Res.

[33] Lee SK, Han YM, Yun J, Lee CW, Shin DS, Ha YR, Kim J, Koh JS, Hong SH, Han DC (2012). Phosphatase of regenerating liver-3 promotes migration and invasion by upregulating matrix metalloproteinases-7 in human colorectal cancer cells. Int J Cancer.

[34] Ochiai H, Nakanishi Y, Fukasawa Y, Sato Y, Yoshimura K, Moriya Y, Kanai Y, Watanabe M, Hasegawa H, Kitagawa Y (2008). A new formula for predicting liver metastasis in patients with colorectal cancer: immunohistochemical analysis of a large series of 439 surgically resected cases. Oncology.

[35] Fang YJ, Lu ZH, Wang GQ, Pan ZZ, Zhou ZW, Yun JP, Zhang MF, Wan DS (2009). Elevated expressions of MMP7, TROP2, and survivin are associated with survival, disease recurrence, and liver metastasis of colon cancer. Int J Color Dis.

[36] Wagenaar-Miller RA, Gorden L, Matrisian LM (2004). Matrix metalloproteinases in colorectal cancer: is it worth talking about?. Cancer Metastasis Rev.

[37] Shen F, Cai WS, Li JL, Feng Z, Liu QC, Xiao HQ, Cao J, Xu B (2014). Synergism from the combination of ulinastatin and curcumin offers greater inhibition against colorectal cancer liver metastases via modulating matrix metalloproteinase-9 and E-cadherin expression. Onco Targets Ther.

